# An automatic laryngoscopic image segmentation system based on SAM prompt engineering: from glottis annotation to vocal fold segmentation

**DOI:** 10.3389/fmolb.2025.1616271

**Published:** 2025-07-10

**Authors:** Yucong Zhang, Yuchen Song, Juan Liu, Ming Li

**Affiliations:** ^1^ School of Computer Science, Wuhan University, Wuhan, China; ^2^ Suzhou Municipal Key Laboratory of Multimodal Intelligent Systems, Digital Innovation Research Center, Duke Kunshan University, Kunshan, China; ^3^ School of Artificial Intelligence, Wuhan University, Wuhan, China

**Keywords:** medical image analysis, laryngoscope, prompt engineering, segment anything model, vocal fold segmentation

## Abstract

**Introduction:**

Laryngeal high-speed video (HSV) is a widely used technique for diagnosing laryngeal diseases. Among various analytical approaches, segmentation of glottis regions has proven effective in evaluating vocal fold vibration patterns and detecting related disorders. However, the specific task of vocal fold segmentation remains underexplored in the literature.

**Methods:**

In this study, we propose a novel automatic vocal fold segmentation system that relies solely on glottis information. The system leverages prompt engineering techniques tailored for the Segment Anything Model (SAM). Specifically, vocal fold-related features are extracted from U-Net-generated glottis masks, which are enhanced via brightness contrast adjustment and morphological closing. A coarse bounding box of the laryngeal region is also produced using the YOLO-v5 model. These components are integrated to form a bounding box prompt. Furthermore, a point prompt is derived by identifying local extrema in the first derivative of grayscale intensity along lines intersecting the glottis, offering additional guidance on vocal fold locations.

**Results:**

Experimental evaluation demonstrates that our method, which does not require labeled vocal fold training data, achieves competitive segmentation performance. The proposed approach reaches a Dice Coefficient of 0.91, which is comparable to fully supervised methods.

**Discussion:**

Our results suggest that it is feasible to achieve accurate vocal fold segmentation using only glottis-based prompts and without supervised vocal fold annotations. Extracted features on the resulting masks further validate the effectiveness of the proposed system. To encourage further research, we release our code at: https://github.com/yucongzh/Laryngoscopic-Image-Segmentation-Toolkit.

## 1 Introduction

In today’s society, communication is an important part of people’s life and work (Rodero, 2018; Vieira et al., 2020). Correctly producing voice signals is critical to transmitting information effectively and accurately in verbal communication (Diehl and McDonald, 1956). The voice production process has several main steps, but phonation is the most important step among all (Cataldo et al., 2013). Phonation happens when the vocal folds in the larynx vibrate as air from the lungs passes through them (Gordon and Ladefoged, 2001), so the observation and study of vocal folds vibration patterns are substantially helpful for the diagnosis of phonation-related diseases. Healthy vocal folds are symmetrical and vibrate periodically when producing sound. In contrast, the abnormality in the periodic vibration and the asymmetrical shape results in vocal disorders (Herzel et al., 1994).

In clinical diagnosis, laryngeal imaging techniques are used for quantitative measurement and interpretation of the vocal fold vibration (Hirose, 1988; Sung et al., 1999). State-of-the-art technology is the laryngeal high-speed video endoscopy (HSV) that enables a real-time recording of the vocal fold vibration (Lohscheller et al., 2007). In common practice, the diagnosis of vocal disorders is based on doctors’ subjective analysis of HSV recordings (Verikas et al., 2009). However, this subjective observation and evaluation of vibration period, vocal fold symmetry, the degree of vocal fold closure time, and many other features are often time-consuming, experience-based, and error-prone (Ghasemzadeh and Deliyski, 2022). To alleviate the limitations brought by subjective diagnosis, objective features are important for a quantitative analysis of HSV recordings.

Therefore, many studies have focused on methods that can automatically extract features to assist clinicians. Glottal area waveform (GAW) (Noordzij and Woo, 2000) is the most widely used one, which shows the changes of the glottal area through time. This feature is able to provide useful information for analyzing the periodic patterns of vocal fold oscillation and the condition of glottis closure. To obtain a better GAW, it requires an accurate segmentation of the glottal area. Traditional methods, like thresholding (Yan et al., 2006), watershed algorithms (Osma-Ruiz et al., 2008), and active contour models (Karakozoglou et al., 2012), utilize physical features to segment the glottal area. Nevertheless, if the recording condition changes, those methods that depend on physical features might not work well.

In recent years, deep supervised learning models, particularly those based on U-Net architectures (Ronneberger et al., 2015; Huang et al., 2020; Xu et al., 2023; Li et al., 2024; Huang L. et al., 2024), have achieved notable success in medical image segmentation. While these models have been extensively applied in areas such as lung CT image segmentation, there has been comparatively less research on the segmentation of the human larynx. Most studies in this domain have focused on glottis segmentation for quantitative analysis, with Derdiman and Koc (2021), Zhang et al. (2024) validating the effectiveness of U-Net on glottis images, and Lee et al. (2023) enhancing U-Net with a dual-attention mechanism to improve segmentation accuracy. However, vocal fold segmentation remains an underexplored area due to the variability in vocal fold shape, color, and size, and the indistinct boundaries that challenge both manual annotation and automated segmentation. Although several open-source laryngoscopic datasets exist for glottis annotation, almost none of them provide vocal fold annotations, with the exception of Fehling et al. (2020).

The scarcity of annotated data is a common challenge in medical image segmentation, exacerbated by patient privacy concerns. Recent studies have turned toexplored the Segment Anything Model (SAM) (Kirillov et al., 2023) for zero-shot image segmentation, as it does not require target annotations during inference. due to its ability to generalize across diverse tasks using only generic prompts, eliminating the need for task-specific annotations during inference. SAM accepts various prompts as inputs and generates corresponding segmentation masks. In this work, we aim to design prompts that leverage glottis annotations as prior knowledge for SAM, exploring its potential for vocal fold segmentation. The main contributions of our work are outlined as follows.

1. The proposed system utilizes only glottis information to segment vocal folds in an unsupervised manner. This approach addresses the scarcity of open-source vocal fold annotation data and reduces the labor costs associated with manual vocal fold annotation.
2. We introduce a prompt engineering method to extract both bounding box and point prompts for SAM to segment vocal folds. To our knowledge, this is the first exploration of SAM’s segmentation capabilities on human larynx.
3. On the open-sourced public dataset (Fehling et al., 2020), our proposed system, trained solely on glottis annotation data, achieves performance comparable to supervised methods trained directly on vocal fold annotation data.
4. We extract potential useful metrics from the vocal fold masks using our prompting method, which show abnormal signs of patients.

The following article is formed as follows. In Section 2, we introduce related works on both vocal folds segmentation and SAM. In Section 3, we provide a comprehensive introduction of our system, including laryngeal prompt engineering for SAM and vocal folds mask inference with SAM. Section 4 shows our experimental settings and results. To further demonstrate the effectiveness of our system, parameter tuning and ablation studies are also discussed in Section 4. In Section 5, we discuss the limitation of our prompting methods and include our future works. In the end, we summarize our paper in Section 6.

## 2 Related works

### 2.1 Vocal fold segmentation

In addition to glottis segmentation, the segmentation of vocal folds plays a crucial role in the clinical diagnosis of laryngeal diseases. However, vocal fold segmentation is inherently more challenging than glottis segmentation. While the glottis typically features well-defined and easily discernible boundaries, vocal folds exhibit significant variability in shape, size, and color across individuals, along with complex and less distinct boundaries. Despite its importance and difficulty, there are only a few existing works focusing on vocal fold segmentation. Fehling et al. (2020) introduced a modified U-Net model called Convolutional Long Short-Term Memory Network (CLSTM), which incorporates Long Short-Term Memory Networks (LSTM) and Gated Recurrent Units (GRU) as inter-layers to propagate temporal information across the network (Fehling et al., 2020). The authors also provided an open-source dataset containing annotations for the glottis as well as the left and right vocal folds, which, to the best of our knowledge, is one of the few publicly available datasets that include vocal fold labels. Their model achieved mean Dice coefficients of 0.85, 0.91, and 0.90 for the glottis, right vocal fold, and left vocal fold, respectively, on their test set, demonstrating the efficacy of supervised methods. However, several limitations still persist. First, the annotation of masks for both the vocal folds and glottis requires manual input for each image. While glottis labels can be generated with relative ease, the annotation of vocal fold masks is labor-intensive and time-consuming. Furthermore, the robustness of the model remains unverified due to the limited quantity of labeled data (13,000 images derived from 130 high-speed video recordings using similar laryngoscopes) and the lack of additional public datasets. To address these challenges, this work seeks to develop methods for vocal fold segmentation that do not rely on fully supervised learning models.

### 2.2 Segment anything model on medical images

The Segment Anything Model (SAM) is a recently introduced deep learning-based segmentation model renowned for its strong generalization capabilities (Kirillov et al., 2023). SAM exhibits remarkable potential in zero-shot segmentation tasks, requiring only minimal input prompts such as bounding boxes, points, text, or even no prompts at all. Given the increasing demand for medical image segmentation, several studies have evaluated SAM’s performance across various types of medical images, including Computed Tomography (CT), Magnetic Resonance Imaging (MRI), and endoscopy. The most straightforward approach involves directly applying the pre-trained SAM to different segmentation tasks, which has demonstrated robust annotation capabilities in certain domains (Hu et al., 2023; Mohapatra et al., 2023). However, SAM’s performance has been found to be suboptimal compared to traditional segmentation models in specific contexts (Deng et al., 2023). Comparative analyses of different medical imaging modalities suggest that SAM’s effectiveness is influenced by factors such as task complexity, image dimensionality, target region size, and the contrast between the target and background (Deng et al., 2023; He et al., 2023). Consequently, numerous researchers have focused on fine-tuning SAM for particular segmentation tasks. For example, MedSAM is a SAM-based model fine-tuned using over 1 million image-mask pairs spanning 10 modalities (Ma et al., 2024), resulting in significant improvements in universal medical image segmentation. However, the substantial data requirements for effective fine-tuning, coupled with the limited availability of open-source medical images, have led many researchers to explore prompt engineering for the pre-trained SAM (Yu et al., 2023; Huang J. et al., 2024). This approach has also yielded promising results in zero-shot image segmentation. Nevertheless, limited research has focused on the human larynx, which motivates the application of a similar strategy for vocal fold segmentation. In this work, we aim to design effective prompts derived from glottis information to enable SAM to segment vocal folds.

## 3 Materials and methods

### 3.1 Overview

**FIGURE 1:**
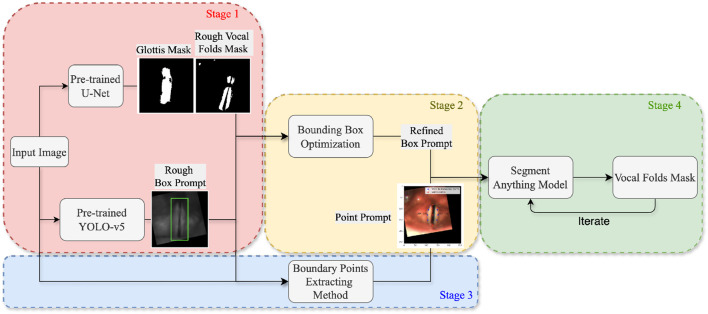
Flowchart illustrating the main components of the proposed system.

As illustrated in Figure 1, the system architecture comprises four stages. In Stage 1 (described in Section 3.2), a glottis mask and a preliminary bounding box of the vocal folds are obtained through the inference of two pre-trained models: U-Net (Ronneberger et al., 2015) and YOLO-v5 (Jocher et al., 2022). In Stage 2 (described in Section 3.3), a more accurate bounding box is derived by applying multiple computer vision techniques, utilizing the glottis masks and the estimated bounding boxes generated in the previous stage. Stage 3 (described in Section 3.4) involves the extraction of points corresponding to the outer boundaries of the vocal folds. Finally, in Stage 4 (described in Section 3.5), the refined bounding box and the edge points obtained in the preceding stages are provided as box and point prompts to the Segment Anything Model (SAM) (Kirillov et al., 2023), along with the mask generated in the previous iteration. The core objective of the proposed method is to extract bounding boxes and points that effectively prompt SAM, thereby facilitating high-quality segmentation of the vocal folds.

### 3.2 Glottis mask extraction

Previous works on glottis segmentation have predominantly employed supervised learning methods, where a segmentation model is trained on manually labeled datasets. Researchers in Gómez et al. (2020) demonstrated that, with a basic U-Net model and sufficient glottis mask annotations, successful segmentation of the glottal area can be achieved. In this work, we use a pre-trained U-Net model (Zhang et al., 2024) using the same methodology described in Gómez et al. (2020). The pre-trained model accepts a laryngoscopic image as input and produces raw output values (logits) for each pixel. These logits are subsequently converted into probabilities ranging from 0 to 1 through the application of the sigmoid activation function. A threshold of 0.5 is then applied to generate a binarized mask image, labeling the glottis region.

**FIGURE 2:**
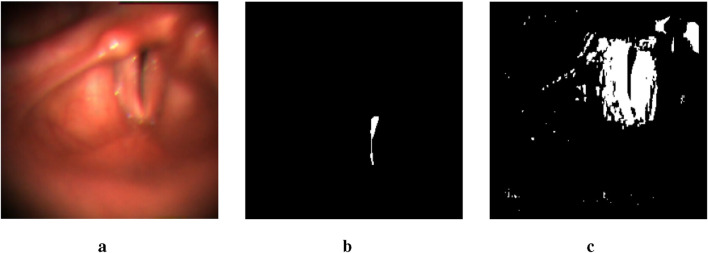
An example of two output masks generated by the pre-trained U-Net model. The image in the middle is a glottis mask. The one on the right is a mask obtained by setting the threshold to be lower than 1e-17. **(a)** Input image. **(b)** thres.>0.5. **(c)** thres.<1e-17.

While the glottal area is relatively easier to annotate compared to the vocal folds, which has resulted in fewer studies on vocal fold segmentation, we make a surprising discovery. By lowering the threshold from 0.5 to a very small value, the glottis segmentation mask generated by the U-Net model includes a rough segmentation mask of the vocal folds. As illustrated in Figure 2, despite the presence of numerous noisy points, the mask image contains a large white region near the glottis that corresponds to part of the vocal fold area. This observation enables us to locate the region of the vocal folds more accurately.

### 3.3 Bounding box prompt extraction

**FIGURE 3:**
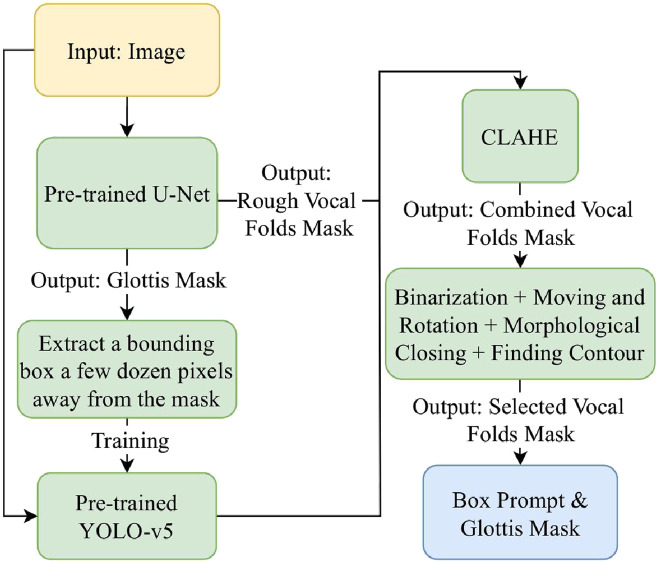
The workflow of the box prompt extraction stage.

**FIGURE 4:**
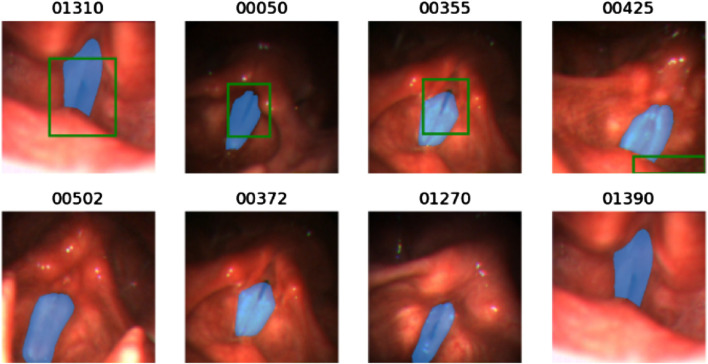
Illustration of some bad cases of the bounding box generated by the pre-trained YOLO-v5 model. For each image, the blue mask is the ground truth mask and the green bounding box is the output of the pre-trained YOLO-v5 model. The below four figures show that the pre-trained YOLO-v5 model fails to detect the glottal area.

As Figure 3 illustrates, we first train a YOLO-v5 model on HSV images with a bounding box extracted a few dozen pixels away from the glottis mask predicted by the U-Net model (Zhang et al., 2024). Therefore, it is a rough estimation of the vocal fold area, which takes advantage of the physical structure of the human larynx. However, since the training data provides only a rough estimation of the target region, the object detection capability of the trained model is insufficient, leading to bad cases. Figure 4 shows some of the bad cases, including no bounding box, wrong bounding box, bounding box that is too small for the target area, etc. In order to provide a more accurate bounding box, we also take advantage of the U-Net mask and apply some traditional computer vision methods to process the mask and extract the information of vocal folds in it.

**FIGURE 5:**
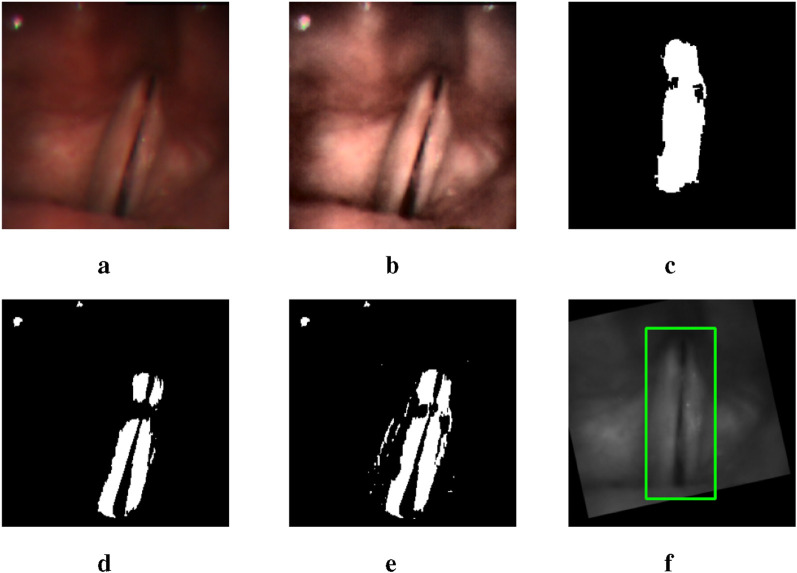
A group of images illustrating the outputs of different intermediate steps and the final bounding box obtained: **(a)** the original input image, **(d)** the U-Net mask generated using the original image, **(b)** the CLAHE processed image, **(e)** the combination of two mask images generated by the original image and the processed image respectively, **(c)** the selected mask with rotation and moving, and **(f)** the final bounding box.

By observing the U-Net mask, we find that the white points in the mask image often correspond to the areas with high contrast between light and dark in the original image. Therefore, we apply the Contrast-Limited Adaptive Histogram Equalization (CLAHE) method to the image (Pizer et al., 1987). As Figure 5 shows, after processing the input image, the output mask contains more complete information of vocal folds, though along with more noisy points. Thus, in the mask image obtained by the original image, we only replace the part in the bounding box region obtained by the YOLO model with the mask image obtained by the CLAHE processed image. In this way, we can avoid adding noise in non-target areas.

Then we find the middle point of the glottis mask and the connection line between its upper and lower points. By moving and rotating, we make the connection line vertical and the middle point of the glottis in the middle of the image. This processing can reduce the error caused by different shooting angles of the camera, as the bounding box extracted on the rotated and moved image has a more precise estimation, narrowing the non-target area in the image.

In order to extract the mask that represents the vocal fold area in the mask image, we use the following methods. First, we apply the morphological closing method to the mask image to connect separate but close contours (Salembier et al., 1998). To identify the contour most relevant to the vocal fold region, we incorporate the previously generated glottis mask into the mask image. The contour that encompasses the integrated glottis mask is then selected as the extracted mask (Suzuki et al., 1985). Figure 5c is an example of the mask extracted after applying the methods.

Finally, we rotate and move the extracted mask using the same rotation and moving matrix and extract a bounding box accordingly. The bounding box is then averaged with the one predicted by the YOLO-v5 model. This final bounding box serves as the box prompt provided to the SAM.

### 3.4 Point prompt extraction

**FIGURE 6:**
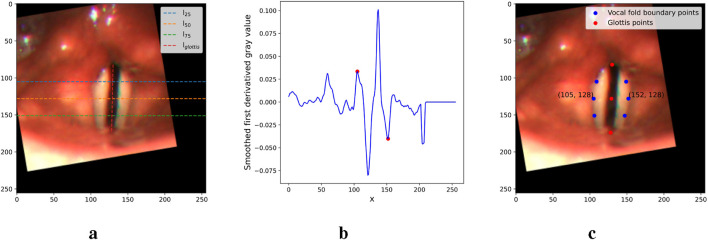
Figures illustrating the main steps in the point prompt extraction. **(a)** Critical lines. **(b)** First derivative of $l_{50}$ . **(c)** Point prompts.

To extract the point prompts, we first connect the top, middle, and bottom points of the glottis with a vertical line, $l_{\mathrm{glottis}}$ , as shown in Figure 6a. Next, we define three horizontal lines, $l_{25}$ , $l_{50}$ , and $l_{75}$ , which are orthogonal to the vertical line. Specifically, these three lines pass through the three quadrisection points of the vertical line respectively. For each of these lines, we calculate the first derivative of the gray values of the pixels along the line, and we apply a smoothing function to minimize the impact of local extrema. Figure 6b illustrates an example of the smoothed values along $l_{50}$ . To identify the left and right boundary points of the vocal folds from the plot, we select the first local maximum to the left of the glottis region (corresponding to the left boundary) and the first local minimum to the right of the glottis region (corresponding to the right boundary), as indicated by the two red points in the example plot. This approach works because the gray values increase rapidly from shadow to vocal fold surface on the left boundary, and decrease sharply from surface to shadow on the right boundary. Figure 6c shows the six extracted points along the three horizontal lines in blue, as well as the three glottis points in red. Together, these nine points represent the extracted point prompts.

### 3.5 Inference with SAM

SAM is a prompt-based model that takes an image and prompts including boxes, points, texts, and even rough masks as inputs (Kirillov et al., 2023). As for the architecture, it is a transformer-based model consisting of three main components: an MAE pre-trained Vision Transformer (ViT) based image encoder that encodes the input image into features, a prompt encoder integrating prompts provided by users, and a mask decoder that generates a segmentation result by mapping the image embedding, prompt embeddings, and an output token to a mask.

In this research, we utilize the original pre-trained SAM proposed by Kirillov et al. (2023) and provide the box and points prompts extracted in previous stages. In addition, inspired by studies on the prompt engineering of the SAM and utilizing the feature of the model that receives a rough mask as input, we try one or more iterations of SAM inference. The method can be explained as follows. In the first iteration, we input a point prompt and obtain a rough mask (logits). In the following iterations, the inputs are the point prompt and the logits. In the final iteration, we add a box prompt as the third input and obtain the mask as the final output.

## 4 Experimental results

### 4.1 Datasets

We use two open-source laryngoscopic image datasets for different purposes in the evaluation. This study (usage of existing databases) is approved by the Duke Kunshan University Institutional Review Board (IRB No. 2024ML023).

#### 4.1.1 Benchmark for automatic glottis segmentation

The first is the Benchmark for Automatic Glottis Segmentation (BAGLS), a large dataset of endoscopic high-speed video with 59,250 frame-wise glottis annotations (Gómez et al., 2020). The frames are extracted from 640 healthy and disordered larynx recordings that were recorded under varying conditions (illumination, image resolution, endoscopy types, etc.). The ground truth glottis masks were annotated by clinical experts. We use the same recipe as is described in Gómez et al. (2020) to train the U-Net model for glottis segmentation, and we train the YOLO-v5 model for rough vocal folds bounding box extraction.

#### 4.1.2 Fehling’s dataset

The second dataset is provided by Fehling et al. (2020), which contains 13,000 frames extracted from 130 HSV recordings, 100 images each. The recordings cover both healthy and disordered cases, such as polyps, carcinomas, and dysphonia. The ground truth masks are manually annotated and contain left and right vocal folds and glottis labels. In our work, we adjust the parameters of our system on the training set and test our performance on the test set, using the same dataset split setting described by Fehling et al. (2020).

### 4.2 Model efficiency analysis

Table 1 summarizes the computational complexity, parameter count, and inference time of the main components in our method. Notably, YOLO and U-Net demonstrate relatively low inference times (9.95 ms and 15.33 ms, respectively), making them efficient for feature extraction. While SAM involves higher computational demands due to its extensive pre-trained capabilities, its integration with lightweight modules ensures that the overall pipeline remains practical for real-time applications.

**TABLE 1:** Model complexity, number of parameters, and inference time for different modules.

Model	FLOPs (G)	Parameters (M)	Average inference time (ms)
YOLO (Object Detection)	2.52	7.01	9.95
U-Net (Image Segmentation)	109.32	31.04	15.33
SAM	2,730	631.58	636.16

### 4.3 Segmentation metric

To compare with the work of Fehling et al. (2020), we use the same metric called Dice Coefficient (DC) (Dice, 1945) to measure the similarity between the ground truth and the segmentation result. DC metric is calculated by Equation 1.

$$DC\left(x\right)=\frac{2|GT\left(x\right)\cap Seg\left(x\right)|+\epsilon }{|GT\left(x\right)|+|Seg\left(x\right)|+\epsilon },$$ (1)

where *GT(x)* and *Seg(x)* represents the Ground Truth and the segmentation result respectively. The $\epsilon =2.2204\cdot 10^{-16}$ is set to avoid the denominator being zero when there is no intersection due to the possible false segmentation and the complete glottal closure.

### 4.4 Hyper-parameter tuning

We use the following ordered selection strategy to demonstrate the rationality of some methods in the proposed system, and identify the best-performing parameters accordingly, using the training set. As Table 2 illustrates, different thresholds of U-Net outputs for mask generation, the impact of CLAHE processing, and various inference iterations for SAM are evaluated in the experiments. We first choose the best-performing thresholds of the pre-trained U-Net model. The thresholds 1e-19 and 1e-20 reach the best performance among all the thresholds with the powers of 10 ranging from −15 to −21 . This indicates the masks obtained under these thresholds contain the most proper information on vocal fold area for box prompt extraction in the SAM inference stage. Next, a comparative experiment is conducted to prove the effectiveness of the image pre-processing method, CLAHE. The result is consistent with our observation that by applying the brightness contrast enhancement method to the input images, the U-Net model can generate masks containing more information on the vocal fold region. Then we identify the best-performing iteration number for SAM inference and the best threshold using the selected number. According to the previous analysis, the best-performing parameters and methods of the system are YOLO-v5 + U-Net (1e-20) + CLAHE + SAM (2 iterations), and the best Dice score is 0.8227, 0.7883, 0.7776 and 0.7537 for the entire vocal folds, left and right one, and the glottis respectively.

**TABLE 2:** Our proposed segmentation performance on the training set of the Fehling’s Dataset using different hyper-parameters. VF stands for vocal fold. CLAHE stands for the image processing method by Pizer et al. (1987).

Conditions				DC			
YOLO-v5	U-Net (thresholds)	CLAHE	SAM (iterations)	VF	Left VF	Right VF	Glottis
✓	1e-15	✓	1	0.6447	0.6179	0.6139	0.7537
	1e-16			0.7340	0.7000	0.6993	
	1e-17			0.7742	0.7374	0.7367	
	1e-18			0.7981	0.7621	0.7594	
	1e-19			**0.8033**	0.7693	0.7651	
	1e-20			**0.8033**	**0.7697**	**0.7662**	
	1e-21			0.7996	0.7668	0.7622	
	1e-19	×		0.7752	0.7380	0.7382	0.7439
	1e-18			0.7791	0.7423	0.7420	
	1e-19	✓	2	0.8205	0.7870	0.7743	0.7537
	1e-20			**0.8227**	0.7883	**0.7776**	
			3	0.8223	0.7900	0.7753	
			4	0.8211	**0.7901**	0.7734	

Bold values represents the highest values.

**FIGURE 7:**
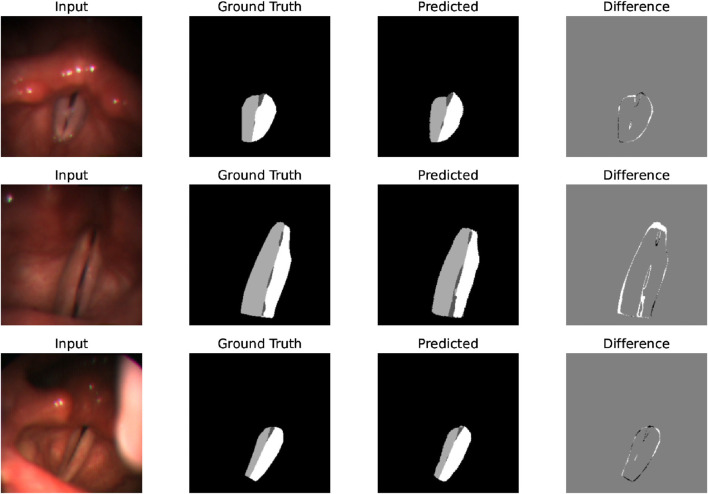
Examples that display the input image, ground truth mask, and predicted mask showing the effectiveness and good performance of the system’s segmentation on laryngoscopic images.

After identifying the best-performing parameters of the system on the training set, using these parameters, we compare the proposed model’s performance of the glottal area, vocal folds area, and left and right vocal fold segmentation with the CLSTM model on the test set. As Table 3 shows, our system has a Dice score of 0.9181 on the vocal fold region, which is very close to the supervised CLSTM model’s performance, 0.9218. For the glottis segmentation, our supervised U-Net model reaches a higher Dice score of 0.8548 than the CLSTM model. However, since we simply separate the entire vocal fold mask into two-halves based on the midline, the Dice score of each side of the vocal fold is relatively lower than the supervised model. Overall, by comparing the Dice score of the completely supervised vocal folds and glottis segmentation model, the result shows the effectiveness and potential of the proposed system based on supervised learning of glottis segmentation, using a series of processing methods and applying the powerful SAM to achieve unsupervised vocal fold segmentation. Figure 7 clearly illustrates some examples of our approach, displaying input laryngoscopic images with their corresponding ground truth and predicted masks. In each mask, dark grey, light grey, and white regions represent the glottis, left and right vocal fold masks respectively.

**TABLE 3:** Segmentation performance on the test set of the Fehling’s Dataset. VF stands for vocal fold.

Models	VF	Left VF	Right VF	Glottis
Our proposed unsupervised system with the best-performing parameters	0.9181	0.8930	0.8919	0.8548
Supervised CLSTM system (Fehling et al., 2020)	0.9218	0.9087	0.8988	0.8502

### 4.5 Ablation study on various prompting methods

To prove the effectiveness of the SAM prompt engineering method proposed in this work, we conducted an ablation study using different SAM prompt conditions. Table 4 displays the segmentation performance under these conditions. When only the extracted box prompts or the point prompts are provided, the average Dice score of the vocal fold masks significantly drops to 0.5730 and 0.1862, respectively. For segmentation without any prompt, we utilize the “segment anything” mode of SAM, which performs zero-shot mask generation by taking only the input image. For each generated mask, the model also outputs an IOU score. Accordingly, we select the mask with the highest IOU score as the final segmentation result in the condition without any prompt, yielding an even lower Dice score. This comparison demonstrates that our prompt engineering method, which combines both box and point prompts, leads to a significant performance improvement.

**TABLE 4:** Segmentation performance under different SAM prompt conditions on the test set of the Fehling’s Dataset. VF stands for vocal fold.

SAM prompt conditions	VF	Left VF	Right VF
Our prompt engineering method (Points prompt + Box prompt+2 iterations)	**0.9181**	**0.8930**	**0.8919**
Box prompt only	0.5730	0.5346	0.5607
Point prompt only	0.1862	0.1760	0.1856
Without any prompt	0.1104	0.1069	0.1023

Bold values represents the highest values.

### 4.6 Performance on Segment Anything Model 2

In the course of our research, the involved version of SAM was released (Ravi et al., 2024), which is called SAM2. It introduces a unique memory bank and memory attention design, which together enable robust video analysis. The SAM2 can propagate through sequential frames by using only one prompt for the initial frame, subsequently tracking and segmenting the target object. Due to the structural and prompting similarities between SAM and SAM2, we also evaluate the effectiveness of our proposed prompt engineering method on SAM2. Similar with the experiment on SAM, multiple experimental conditions are conducted on the test set from Fehling et al. As shown in Table 5, we first leveraged SAM2’s video propagation capability by prompting only the first frame. Under this condition, when prompted with the proposed engineering method—first by providing the extracted points prompt and subsequently applying the same points prompt alongside the obtained box prompt in the second iteration—the model achieved an average Dice score of 0.9071 for the vocal fold mask. In comparison, using only bounding boxes or the points prompt resulted in lower scores of 0.7560 and 0.8570, respectively. We then applied SAM2’s “segment anything” mode without any prompts, and we obtain a substantially lower score. Since SAM2 keeps its original functionality for users to provide prompts for each frame of the video, we use our proposed prompts on every frame to test the performance. It turns out that, in conjunction with the propagation function, the Dice score for the vocal fold mask further improved to 0.9092.

**TABLE 5:** Segmentation performance under different SAM2 prompt conditions on the test Set of the Fehling’s Dataset (Fehling et al., 2020). VF stands for vocal folds.

SAM2 prompt conditions		VF	Left VF	Right VF
Prompt each frame with the proposed method		**0.9092**	**0.8826**	**0.8823**
Prompt thefirst frame only	The proposed method	0.9071	0.8813	0.8787
	Box prompt only	0.7560	0.7329	0.7095
	Point prompt only	0.8570	0.8385	0.8199
	“Segment anything” mode	0.0941	0.0678	0.0951

Bold values represents the highest values.

Comparison of the four prompting conditions for a single-frame prompt shows the efficacy of our prompt engineering method. The table shows the performance gains of 15%, 5%, and 81% over box-prompt-only, points-prompt-only, and no-prompt conditions, respectively. Though lower dice scores are achieved by just using one of the proposed prompts on SAM2, the segmentation performance has been significantly improved comparing to using prompts on the original SAM. From Tables 4, 5, when using box prompt only, the dice score for VF segmentation rises from 0.57 to 0.76. The dice score of using points prompt increases dramatically from 0.19 to 0.86. The results suggest that SAM2 has a stronger learning ability on the target than the original SAM, and our proposed prompting methods works well with SAM2’s video propagation function.

When comparing the dice scores obtained by prompting each frame to those by prompting only the first frame, the former yields a higher score. This result shows that our prompting methods can provide additional information on the target that SAM2 does not capture using its propagation function, further showing the advantages of our proposed prompting methods. Moreover, we observed that the optimal performance of SAM2 on the test data is slightly lower than SAM. This may be attributable to error propagation within the segmentation of some intermediate frames.

Overall, our prompt engineering methods shows great potential on the open-sourced dataset, which can effectively prompt SAM and SAM2 to achieve accurate vocal cord segmentation, outperforming SAM2’s novel function that considers temporal features. We acknowledge SAM2’s impressive video analysis capabilities, future works will further explore SAM2’s potential of only prompting a few or even the initial frame for this task through fine-tuning and adjustments to the pre-trained model.

### 4.7 Application potentials

The use of vocal fold masks facilitates the extraction of more detailed metrics from laryngoscopic videos, which is a significant advancement over the use of glottis masks alone. This section discusses new metrics that could be integrated as additional features, assisting clinicians in a more comprehensive evaluation of vocal fold function.

**FIGURE 8:**
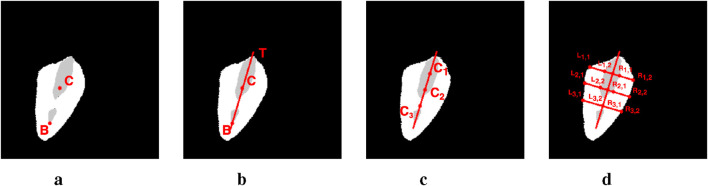
The pipeline of metric computing on image #1149 from the Fehling’s dataset. **(a)** Step 1. **(b)** Step 2. **(c)** Step 3. **(d)** Step 4.

A well-documented correlation exists between the maximal separation of vocal folds and vocal fold paralysis (Inagi et al., 1997). To further this research, scholars have investigated laryngeal features that measure vocal fold separation, both in direct and indirect manners. The GAW and Anterior Glottic Angle Waveform (AGAW) are notable examples (Adamian et al., 2021; Wang et al., 2021; DeVore et al., 2023; DeChance et al., 2024). They are derived from measurements of the glottal area and anterior laryngeal angle in successive video frames. Whilst these provide a general assessment of vocal fold status, they lack the capability to inform on the functionality of individual vocal folds. The primary limitation is their dependence on glottis masks, which are simpler to acquire than vocal fold masks due to variable outlines and colors of the latter. Our methodology seeks to bridge the gap between the widely available glottis masks and the challenging-to-detect vocal fold masks. Following a streamlined labeling procedure adopted by Zhang et al. (2024), our complete labeling process is illustrated in Figure 8. Initially, point D at the bottom and point C , the centroid of the glottis mask, are identified and connected (refer to Figures 8a,b). This line CD hypothesizes the glottis midline, intersecting the glottis mask at point T . Along CD , we locate n equidistant points between D and T (e.g., points $C_{1},C_{2},C_{3}$ ). Lastly, we compute perpendiculars to CD through these equidistant points, which intersect the vocal fold mask at coordinates $L_{i,j},R_{i,j}$ for i=1,2,…,n and j=1,2 .

#### 4.7.1 Vocal fold movement waveform

**FIGURE 9:**
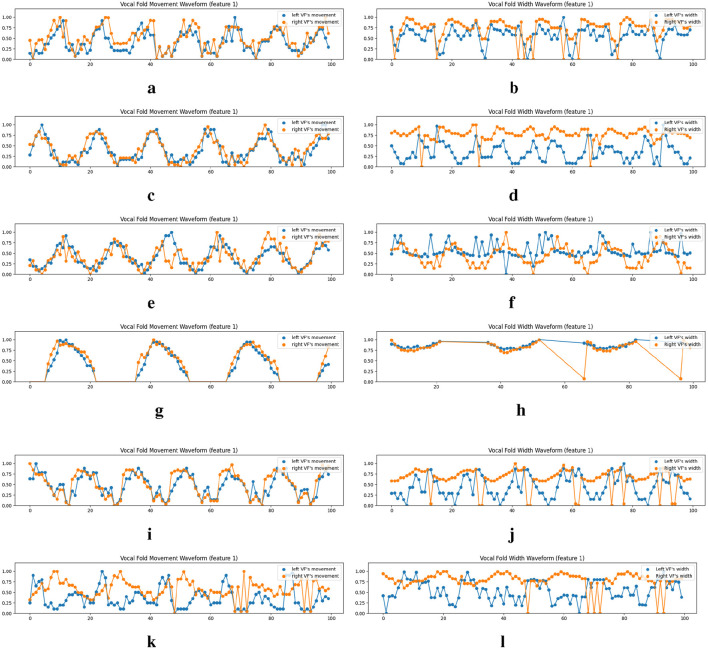
Examples of the vocal fold’s movement and width waveform analysis on the normal cases of the Fehling’s Dataset. VFM and VFW stand for Vocal Fold Movement and Vocal Fold Width respectively. We only show the waveforms for the first equidistance points (feature 1), which are derived from the metrics across $C_{1}$ . For all the waveforms, we collect the points from a total number of 100 consecutive frames from the dataset, forming a video with a length of 4 s for each case. **(a)** VFM on the normal case (video#1). **(b)** VFW on the normal case (video#1). **(c)** VFM on the normal case (video#2). **(d)** VFW on the normal case (video#2). **(e)** VFM on the normal case (video#3). **(f)** VFW on the normal case (video#3). **(g)** VFM on the functional dysphonia case (video#5). **(h)** VFW on the functional dysphonia case (video#5). **(i)** VFM on the paralysis case (video#8). **(j)** VFW on the paralysis case (video#8). **(k)** VFM on the carcinoma case (video#15). **(l)** VFW on the carcinoma case (video#15).

Once the vocal folds are segmented and labeled, we can ascertain the distance of points on each vocal fold from the estimated glottic midline. By averaging the lengths of segments $L_{i,1}C_{i}$ and $L_{i,2}C_{i}$ , we assess the vocal folds’ deviation over time, thereby creating the vocal fold movement waveform. As depicted on the left-hand side of Figure 9, the vocal fold movements of the left and right folds are extracted for both normative and atypical cases. These visualizations vividly demonstrate the phonation cycles of the vocal folds, offering clinicians novel diagnostic perspectives.

#### 4.7.2 Vocal fold width waveform

The width of the vocal folds in each frame is determined by the gap between points $L_{i,1}$ and $L_{i,2}$ for the left vocal fold, with an analogous process for the right vocal fold. This data synthesis results in the vocal fold width waveform (presented on the right-hand side of Figure 9). In this waveform analysis, frames devoid of a glottal area, which complicates accurate vocal fold mask prediction, are excluded, given our method’s substantial reliance on the glottis mask. A waveform comparison reveals greater width stability in patients with functional dysphonia or paralysis, in contrast to the fluctuations captured by the vocal fold movement waveform. These findings may be indicative of vocal fold conditions, providing clinicians with valuable diagnostic information. Additionally, for the carcinoma case shown in Figures 9k,l, the non-periodic nature of the vocal fold width waveform might reveal insights into its vocal fold irregularities.

## 5 Discussion

Our system has achieved a high Dice coefficient on the test dataset of Fehling’s dataset, demonstrating the potential of the SAM prompt engineering method. Nonetheless, it is important to recognize the system’s limitations. Chiefly, the absence of publicly accessible annotated laryngoscopic image datasets has prevented extensive testing under variable conditions, such as different laryngoscope types or lighting environments. Further, within the Fehling dataset, there are cases exhibiting low Dice scores. Upon review, it is evident that our method’s dependence on segmented glottis masks presents challenges in the event of glottis closure. Additionally, the segmentation performance of SAM deteriorates with poor lighting and low-contrast images.

Future research will pursue the integration of our framework with SAM2 for vocal fold segmentation in laryngeal videos. We argue that for video-based inference, SAM2 is better suited than the original SAM, a detail expounded upon in Section 4.6. The incorporation of a memory bank and propagation function in tandem with our initial prompts has yielded superior segmentation outcomes. Current methodology involves straightforward frame-by-frame prompting while maintaining SAM2’s underlying functionality. Prospective enhancements to SAM2’s performance could be achieved through a novel teacher-student model, entailing the fine-tuning of the pre-trained SAM2 with our specialized prompts on a more diversified vocal fold dataset.

## 6 Conclusion

In this work, we developed an automatic laryngoscopic image segmentation system that leverages glottis data for vocal fold segmentation using prompt engineering techniques tailored for the Segment Anything Model (SAM). We initially discover an unexpected utility of low-threshold U-Net outputs in capturing vocal fold information. Then, by using this information, we obtain the bounding box prompt through brightness contrast enhancement and morphological closing with the coarse bounding box of the larynx region generated by the YOLO-v5 model. In addition, we extract vocal fold boundary points as the point prompt by identifying the local extrema of the first derivative of the gray-scale intensity along lines intersecting the glottis. Experimental results demonstrate that our system achieves superior segmentation performance on the vocal fold segmentation task, with results comparable to those of the supervised model. In the end, we show the potential application of our proposed method. We introduce metrics extracted from the vocal folds’ masks that are potentially useful to diagnosis, which cannot be derived from the glottis masks alone.

## Data Availability

The datasets presented in this study can be found in online repositories. The names of the repository/repositories and accession number(s) can be found below: https://zenodo.org/records/3603185.
